# Supplementary material for: MRI-Based Radiomics Input for Prediction of 2-Year Disease Recurrence in Anal Squamous Cell Carcinoma
Source: Cancers (Basel). 2021 Jan 7;13(2):193. doi: 10.3390/cancers13020193 (PMC7827348; doi:10.3390/cancers13020193)
Supplement: Supplementary file 1 [file cancers-13-00193-s001.pdf]

Supplementary Materials: MRI-Based Radiomics Input for Prediction of 2-Year Disease Recurrence in Anal Squamous Cell Carcinoma

Nicolas Giraud, Olivier Saut, Thomas Aparicio, Philippe Ronchin, Louis-Arnaud Bazire, Emilie Barbier, Claire Lemanski, Xavier Mirabel, Pierre-Luc Etienne, Astrid Lièvre, Wulfran Cacheux, Ariane Darut-Jouve, Christelle De la Fouchardière, Arnaud Hocquelet, Hervé Trillaud, Thomas Charleux, Gilles Breysacher, Delphine Argo-Leignel, Alexandre Tessier, Nicolas Magné, Meher Ben Abdelghani, Côme Lepage and Véronique Vendrely

Supplementary Table 1. Complete list of variables tested in the model's creation

**Clinical and treatment-related data**	Age
	Sex
	HIV status
	Tumor length
	T-stage
	N-stage
	Pre-rectal septum infiltration
	Use of panitumumab
	Total radiation dose
	Chemo-radiotherapy duration
	Type of chemotherapy used
**Radiomic variables (as per the PyRadiomics library)**	original_shape_Elongation
	original_shape_Flatness
	original_shape_LeastAxisLength
	original_shape_MajorAxisLength
	original_shape_Maximum2DDiameterColumn
	original_shape_Maximum2DDiameterRow
	original_shape_Maximum2DDiameterSlice
	original_shape_Maximum3DDiameter
	original_shape_MeshVolume
	original_shape_MinorAxisLength
	original_shape_Sphericity
	original_shape_SurfaceArea
	original_shape_SurfaceVolumeRatio
	original_shape_VoxelVolume
	original_firstorder_10Percentile
	original_firstorder_90Percentile
	original_firstorder_Energy
	original_firstorder_Entropy
	original_firstorder_InterquartileRange
	original_firstorder_Kurtosis
	original_firstorder_Maximum
	original_firstorder_MeanAbsoluteDeviation
	original_firstorder_Mean
	original_firstorder_Median
	original_firstorder_Minimum
	original_firstorder_Range
	original_firstorder_RobustMeanAbsoluteDeviation
	original_firstorder_RootMeanSquared
	original_firstorder_Skewness
	original_firstorder_TotalEnergy
	original_firstorder_Uniformity

	original_firstorder_Variance
	original_glcm_Autocorrelation
	original_glcm_JointAverage
	original_glcm_ClusterProminence
	original_glcm_ClusterShade
	original_glcm_ClusterTendency
	original_glcm_Contrast
	original_glcm_Correlation
	original_glcm_DifferenceAverage
	original_glcm_DifferenceEntropy
	original_glcm_DifferenceVariance
	original_glcm_JointEnergy
	original_glcm_JointEntropy
	original_glcm_Imc1
	original_glcm_Imc2
	original_glcm_Idm
	original_glcm_Idmn
	original_glcm_Id
	original_glcm_Idn
	original_glcm_InverseVariance
	original_glcm_MaximumProbability
	original_glcm_SumEntropy
	original_glcm_SumSquares
	original_glrlm_GrayLevelNonUniformity
	original_glrlm_GrayLevelNonUniformityNormalized
	original_glrlm_GrayLevelVariance
	original_glrlm_HighGrayLevelRunEmphasis
	original_glrlm_LongRunEmphasis
	original_glrlm_LongRunHighGrayLevelEmphasis
	original_glrlm_LongRunLowGrayLevelEmphasis
	original_glrlm_LowGrayLevelRunEmphasis
	original_glrlm_RunEntropy
	original_glrlm_RunLengthNonUniformity
	original_glrlm_RunLengthNonUniformityNormalized
	original_glrlm_RunPercentage
	original_glrlm_RunVariance
	original_glrlm_ShortRunEmphasis
	original_glrlm_ShortRunHighGrayLevelEmphasis
	original_glrlm_ShortRunLowGrayLevelEmphasis
	original_glszm_GrayLevelNonUniformity
	original_glszm_GrayLevelNonUniformityNormalized
	original_glszm_GrayLevelVariance
	original_glszm_HighGrayLevelZoneEmphasis
	original_glszm_LargeAreaEmphasis
	original_glszm_LargeAreaHighGrayLevelEmphasis
	original_glszm_LargeAreaLowGrayLevelEmphasis
	original_glszm_LowGrayLevelZoneEmphasis
	original_glszm_SizeZoneNonUniformity
	original_glszm_SizeZoneNonUniformityNormalized
	original_glszm_SmallAreaEmphasis
	original_glszm_SmallAreaHighGrayLevelEmphasis
	original_glszm_SmallAreaLowGrayLevelEmphasis
	original_glszm_ZoneEntropy
	original_glszm_ZonePercentage
	original_glszm_ZoneVariance
	original_gldm_DependenceEntropy

	original_gldm_DependenceNonUniformity
	original_gldm_DependenceNonUniformityNormalized
	original_gldm_DependenceVariance
	original_gldm_GrayLevelNonUniformity
	original_gldm_GrayLevelVariance
	original_gldm_HighGrayLevelEmphasis
	original_gldm_LargeDependenceEmphasis
	original_gldm_LargeDependenceHighGrayLevelEmphasis
	original_gldm_LargeDependenceLowGrayLevelEmphasis
	original_gldm_LowGrayLevelEmphasis
	original_gldm_SmallDependenceEmphasis
	original_gldm_SmallDependenceHighGrayLevelEmphasis
	original_gldm_SmallDependenceLowGrayLevelEmphasis
